# Isolation of Extracellular Vesicles from Biological Fluids via the Aggregation–Precipitation Approach for Downstream miRNAs Detection

**DOI:** 10.3390/diagnostics11030384

**Published:** 2021-02-24

**Authors:** Maria Y. Konoshenko, Evgeniy A. Lekchnov, Olga E. Bryzgunova, Elena Kiseleva, Inna A. Pyshnaya, Pavel P. Laktionov

**Affiliations:** 1 Institute of Chemical Biology and Fundamental Medicine, Siberian Branch, Russian Academy of Sciences, 630090 Novosibirsk, Russia; lekchnov@gmail.com (E.A.L.); olga.bryzgunova@niboch.nsc.ru (O.E.B.); pyshnaya@niboch.nsc.ru (I.A.P.); lakt@niboch.nsc.ru (P.P.L.); 2 Meshalkin Siberian Federal Biomedical Research Center, Ministry of Public Health of the Russian Federation, 630055 Novosibirsk, Russia; 3 The Federal Research Center Institute of Cytology and Genetics, The Siberian Branch of the Russian Academy of Sciences, 630090 Novosibirsk, Russia; elka@bionet.nsc.ru

**Keywords:** extracellular vesicles, microRNA, prostate cancer, polyethylene glycol, dextran blue, isolation from plasma and urine

## Abstract

Extracellular vesicles (EVs) have high potential as sources of biomarkers for non-invasive diagnostics. Thus, a simple and productive method of EV isolation is demanded for certain scientific and medical applications of EVs. Here we aim to develop a simple and effective method of EV isolation from different biofluids, suitable for both scientific, and clinical analyses of miRNAs transported by EVs. The proposed aggregation–precipitation method is based on the aggregation of EVs using dextran blue and the subsequent precipitation of EVs using 1.5% polyethylene glycol solutions. The developed method allows the effective isolation of EVs from plasma and urine. As shown using TEM, dynamic light scattering, and miRNA analyses, this method is not inferior to ultracentrifugation-based EV isolation in terms of its efficacy, lack of inhibitors for polymerase reactions and applicable for both healthy donors and cancer patients. This method is fast, simple, does not need complicated equipment, can be adapted for different biofluids, and has a low cost. The aggregation–precipitation method of EV isolation accessible and suitable for both research and clinical laboratories. This method has the potential to increase the diagnostic and prognostic utilization of EVs and miRNA-based diagnostics of urogenital pathologies.

## 1. Introduction

Extracellular vesicles (EVs) are heterogeneous membrane-coated vesicles released by a variety of cells into the extracellular microenvironment [1]. Depending on their source, biogenesis, and biophysical properties (size and shape), EVs are divided into many species. Exosomes, microvesicles, and apoptotic bodies are most commonly identified [2,3,4]. Current techniques cannot clearly distinguish each type of EV separately. To avoid confusion, we use EVs as a general term when referring to secreted extracellular vesicles less than 200 µm in diameter (excluding large vesicles, like apoptotic bodies, which are precipitated at 17,000 g), according to the recommendations of the International Society for Extracellular Vesicles (ISEV) [5]. EVs have been observed in all biological fluids [6,7]. The EV surface contains membrane-bounded proteins, glycoproteins and lipids, and the internal content consists of structural and functional proteins, enzymes, lipids and peptides of different lengths, and various types of ribonucleic acids (for a review, see [8,9,10,11] and many others). EVs are known to be involved in the regulation of different biological processes and take part in the pathogenesis of many diseases, including oncological, infectious, neurodegenerative, autoimmune, and cardiovascular disorders (see [9,12] for a review). During cancer initiation and progression, exosomes have been implicated in apoptosis and cell cycle regulation, abnormal tumor angiogenesis, proliferation, metastasis, immune system alterations, drug resistance, etc. [13,14]. Thus, circulating EVs may act as sources of biomarkers for different pathologies, including cancer, so a detailed analysis of their content can be used for non-invasive diagnosis, monitoring of the treatment response, and might be relevant in choosing the treatment type for each specific patient. A liquid biopsy based on the detection of extracellular vesicle-packaged miRNA has high potential to be successfully used in clinical cancer diagnostics [10,11,15,16,17]. MiRNAs are small non-coding RNAs that are involved in the regulation of gene expression at the posttranscriptional level, usually by binding to the 3′ untranslated region (3′ UTR) of mRNA, leading to mRNA degradation or the inhibition of translation [18]. It is known that miRNAs regulate the activity of more than 60% of human protein-coding genes [19,20]. Moreover, various disorders, including cancer, alter miRNA expression [21]. A number of miRNAs have been characterized as oncogenes and tumor suppressors in PCa [22,23]. Extracellular vesicles (EVs) and cell-free nucleoprotein complexes carry distinct populations of miRNAs, and the content of these miRNA pools provide a convenient source of diagnostic molecules for non-invasive PCa screening [10,24]. Nevertheless, it is necessary to develop a simple, fast, and efficient isolation method appropriate for clinic research to use EVs as a source of biomarkers. Presently, many different protocols are used in research laboratories for EV isolation. These protocols are based on various approaches, such as ultracentrifugation (UC), which is considered to be the gold standard of EV isolation, micro- and ultrafiltration, gel-filtration, precipitation, affinity bounding, and microfluidics. Moreover, there are commercical kits, based on different isolation techniques ([25] for review). Along with various advantages, each of these methods has disadvantages that significantly affect the isolated fraction and must be taken into account when choosing the isolation method with a specific goal [25]. For example, the gold standard of EV isolation, UC, is laborious and utilizes complex and expensive equipment. Consequently, this method is inappropriate in a clinical setting. EV isolation using commercial kits based on polyethylene glycol (PEG) 6000 precipitation is simple but non-specific and is, thus, unsuitable for protein analysis and is frequently used to co-isolate immune complexes, aggregates of biopolymers, and PCR inhibitors [26]. Thus, the development of simple, effective and cheap methods of EV isolation, suitable for subsequent analyses (RT-PCR, microarray, genome-wide sequencing, and mass spectrometry), is an urgent task in modern molecular biology and diagnostic medicine.

Low contamination of the sample, reproducibility, low cost, isolation from different types of biological fluids, parallel processing of a large number of samples, and speed and ease of analysis are main characteristics of ideal methods for EV isolation. Until now there has beenno such method which is suitable for a comprehensive analysis of EVs and their content. Even if it is not possible to create universal method of EV isolation, then a method for certain scientific and/or medical applications is urgently needed.

Here, we aim to develop a simple and effective method for EV isolation from different biological fluids, suitable for scientific and clinical analyses of EV-transported miRNAs. We suggest that if the particles are preliminarily aggregated using membranotropic bi/polyfunctional reagents, then it will be possible to precipitate those reagents with a significantly lower concentration of PEG. If the concentration of PEG is lower, then there will be fewer impurities, aggregates, immune complexes, etc., in the sediment. At the same time, this approach may increase the selectivity of microvesicle sedimentation as soon as the microvesicles are aggregated/brought together through the interaction of cibacron with the membrane—i.e., membrane-coated particles will be brought closer together [27]. The developed method is a proof of this concept. UC, the most commonly studied method for EV isolation, was used as the control method. Urine and plasma samples of PCa patients and healthy donors were used to develop and assess the effectiveness of the new method. Earlier it was shown, that urine EVs represent effective source of PCa markers superior to clarified urine and plasma [16,17]. The attractiveness of aggregation–precipitation method for prostate cancer diagnosis and prognosis development lies in the anatomical location of the prostate gland, where tumor-specific biomarkers, including miRNAs carried by extracellular vesicles, enter the blood and urine, which is of particular interest for the development of minimally invasive PCa diagnostics.

## 2. Materials and Methods

### 2.1. Chemical and Biochemical Reagents

Tris HCl, GuSCN, MgCl2, HCl, NaOH, CH3COOH, NaHCO3, NaOAc, 2-mercaptoethanol, octanoic acid, isopropanol, and ethanol were acquired from Sigma-Aldrich (St. Louis, MO, USA). NaCl, phosphate-buffered saline (PBS), and Dextran blue 2000 kDa (DEXB) were obtained from Applichem (Darmstadt, Germany), and Merck KGaA (Darmstadt, Germany), respectively. Na2EDTA was obtained from Fluka (Geneva, Switzerland), and PEG20000 was obtained from Loba Chemie (Mumbai, India). EDTA spray-coated vacutainers were obtained from Fisher Scientific (Waltham, MA, USA). Glycogen, the RiboLockRNAse inhibitor, the MMLV reaction buffer, and MMLV reverse transcriptase were acquired from Fermentas (Vilnius, Lithuania). Primers and probes for reverse transcription and TaqMan qPCR (Table 1) and dNTPs were synthesized at the organic synthesis Laboratory of ICBFM SB RAS (Novosibirsk, Russia), and the 10× PCR buffer and Taq DNA polymerase were obtained from BiolabMix (Novosibirsk, Russia). Cel-miR-39-3p was synthesized in the Laboratory of Medicinal Chemistry (ICBFM SB RAS, Novosibirsk, Russia).

### 2.2. Sample Collection

**Figure 1:**
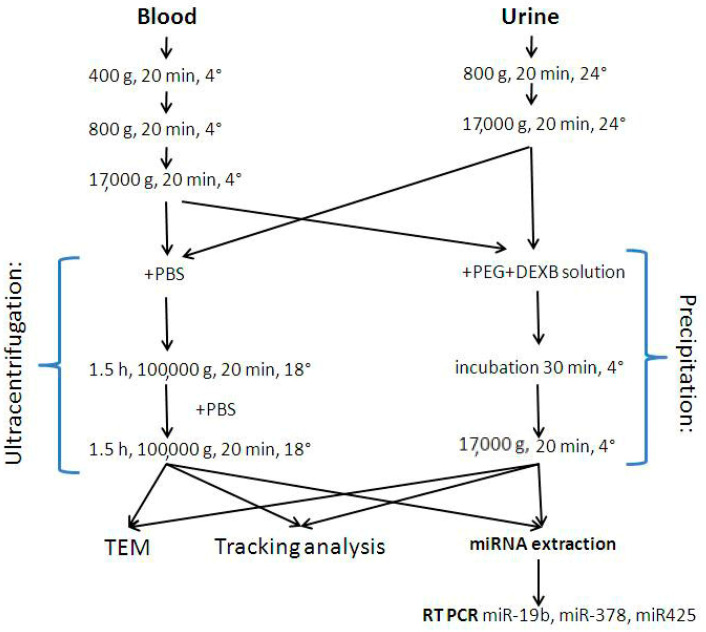
Scheme of the experiment.

The scheme of the study is presented on Figure 1. Blood and urine samples from 12 healthy individuals (male donors with no evidence of prostate diseases, PSA level of <2.8 ng/mL, mean age 53 years, age range 45–60 years old) and 12 PCa patients (male, mean age 67 years, age range 54–77 years old, untreated PCa, T2N0M0) were obtained from the E. Meshalkin National Medical Research Center of the Ministry of Health of the Russian Federation (Novosibirsk, Russia) after approval of the study by the ethics committee (N 15309-01 from 22 December 2008).Written informed consent was provided by both the patients and the healthy volunteers. The study complied with the World Medical Association Declaration of Helsinki regarding ethical conduct of research involving human subjects.

Venous blood was collected in EDTA spray-coated vacutainers, stored at 4 °C, and processed within fourhours. To obtain blood plasma, the samples were sequentially centrifuged at 400× *g* for 20 min and at 800× *g* for 20 min, both at 4 °C. To remove cellular debris, samples were centrifuged at 17,000× *g* at 4 °C for 20 min.

Fresh urine samples were collected in sterile containers. Urinary cells and debris were removed via sequential centrifugation at 400× *g* for 20 min at room temperature and clarified at 17,000× *g* for 20 min at 24 °C to obtain the urine supernatant.

Immediately after urine supernatant and plasma obtainment, the EV fraction was enriched in the resulting samples.

### 2.3. EV Enrichment Procedures

All EVs enrichment procedures were carried out in duplicate.

#### 2.3.1. Ultracentrifugation

A volume of 500 µL of human plasma or 5 mL of human urine was increased to 12 mL with phosphate-buffered saline (PBS), transferred to a 14 mL open top Ultra-ClearTM centrifuge tube (Beckman Coulter, Brea, CA, USA), and centrifuged at 100,000× *g* for 90 min at 18 °C in a Beckman Coulter Optima TM L-90k centrifuge with an SW40Ti rotor (Beckman Coulter). The pellet was washed by resuspending it in 10 mL of PBS followed by centrifugation under the same conditions. Finally, the pellet was resuspended in 500 µL PBS, snap-frozen in liquid nitrogen, and stored at −80 °C.

#### 2.3.2. EV precipitation

Blood plasma

In total, 500 µL of human blood plasma was mixed sequentially with 1.25 mL 1M NaCl, 0.377 mL PBS, 0.124 mL 1M TrisHCl (pH = 7.0), 0.1mL DEXB (0.1 mg/mL), and 150 µL of PEG solution (25% PEG 20000 in 0.01 M PBS, pH = 7.4) via repeated pipetting and incubated for 30 min at 4 °C. Samples were then centrifuged at 17,000× *g* (FA rotor, Eppendorf 5810r) for 20 min. The supernatant was subsequently discarded, and the pellet was resuspended in PBS (500 µL), frozen in liquid nitrogen, and stored at –80 °C for subsequent miRNA isolation or EV analysis.

Urine

We mixed 5 mL of human urine with 0.36 mL 1M NaCl, 0.29 NaHCO3, 0.3 mL DEXB (0.1 mg/mL), and 440 µL PEG solution (25% PEG 20000 in PBS) via repeated pipetting and incubated the samples for 30 min at 4 °C. The samples were then centrifuged at 17,000× *g* (Eppendorf 5810r) for 20 min. The supernatant was discarded, and the pellet was resuspended in PBS (500 µL), frozen in liquid nitrogen, and stored at −80 °C for subsequent RNA extraction or EV analysis.

### 2.4. Transmission Electron Microscopy 

Fresh samples of extracellular vesicles (20 μL) were adsorbed for 1 min on copper grids covered with formvar film and stabilized by carbon. The grids were exposed for 5–10 s on a drop of 0.5% uranyl acetate; then, the excess fluid was removed using filter paper, and the grids were airdried. The size distribution of vesicles was evaluated using 36 TEM images from three healthy donors and three PCa patients (six images per patient, a median of ~39 vesicles per image, no less than 200 per patient). Counting was performed manually; for example, the 31–50 nm group presented vesicles sized ≥30.1 nm but ≤50 nm. Grids were analyzed using a JEM1400 (80 kV, Jeol, Japan) transmission electron microscope supplied with a digital camera Veleta (Olympus SIS, Germany). The measurements were performed using the iTEM (Olympus SIS, Germany) software. TEM imaging was performed at the Microscopy Center of Biological Subjects of ICG SB RAS.

### 2.5. Characterization via Dynamic Light Scattering

The size values of all EV fractions were evaluated using a Zetasizer Nano ZS Plus instrument (Malvern Instruments; Malvern, UK) via dynamic light scattering. 

The samples were measured without further dilution. The obtained data concerning the particle size, i.e., the intensity-based size distribution plots, were expressed as the z-average. Each sample was prepared in triplicate and measured three times at room temperature. The data reported are the average of three measurements on the same sample aliquot. Data were analyzed using the Zetasizer Software to calculate the hydrodynamic diameters of the particles.

### 2.6. miRNA Isolation by the Gu/OcA Protocol 

Before isolation of the miRNA, the blood plasma or urine EV samples were thawed and mixed gently. Gu/OcA miRNA isolation from plasma and urine EVs was performed as described for clarified plasma and urine, respectively [28]. After the addition of the denaturation buffer, synthetic cel-miR-39-3p was spiked into the samples at 5 × 10^7^ copies per isolation. MiRNAs isolations were carried out in duplicate.

### 2.7. RNA Precipitation

To concentrate the miRNA samples after isolation, the procedure of RNA precipitation by isopropanol was performed as described previously in Lekchnov et al. (Lekchnov et al., 2016). To stabilize the miRNA, 1.5 µL of glycogen (20 mg/mL) was added into each tube. Air-dried miRNA pellets were then dissolved in 30 µL of RNAse-free water.

### 2.8. ReverseTranscription and Quantitative RT-PCR

Reverse transcription (RT) on miRNA templates was performed as described by Chen et al. [29]. Primers and probes for reverse transcription and TaqMan qPCR (Table 1) were synthesized in the Laboratory of Medicinal Chemistry (ICBFM SB RAS, Novosibirsk). Each RT reaction was performed in a total volume of 10 µL and contained 2.5 µL of RNA, 25 nM each of miRNA-specific primers, 50 units of MMLV reverse transcriptase (Fermentas, Vilnius, Lithuania), 2 mL of 5× MMLV reaction buffer, and 125 mM of each dNTP. The reaction conditions were as follows: 16 °C for 30 min, 42 °C for 30 min, and 70 °C for 10 min. Samples without RNA templates were used as negative controls.

Real-time PCR was carried out on the LightCycler 480 II detection system (Roche, Switzerland). All reactions were carried out in duplicate in a total volume of 24 µL. Each reaction contained 4 µL of the RT product, 1 unit of Taq DNA polymerase (BiolabMix, Russia), 2.4 µL of 10× PCR buffer (750 mM TrisHCl (pH = 8.8 at 25 °C), 200 mM (NH4)2SO4, 0.1% Tween-20), 3.2 mM MgCl2, 200 mM of each dNTP, 480 nM forward primer, 640 nM universal reverse primer, and 240 nM specific TaqMan probe (Konoshenko et al., 2020). After initial denaturation at 95 °C for 3 min, the reactions were run for 50 cycles at 95 °C for 15 s and at 60 °C for 45 s.

The threshold cycle (Cp) values of miR-19b, miR-378a, and miR-425 were compared in the samples isolated by different protocols from biofluids obtained from the same individuals. These miRNAs were used for analysis, as they were present in sufficient quantities for analysis in both plasma and urine EVs.

### 2.9. Statistical Analysis

Statistical analysis was carried out with the Statistica software using a factorial ANOVA model with the donor’s status, type of biological fluid, and isolation method as factors, followed by a Fisher’s least significant difference (LSD) test. To analyze the diluted miRNA samples, a repeated measures ANOVA was conducted with donor’s status, type, of biological fluid, and extraction method used as factors, followed by a Fisher’s least significant difference (LSD) test. Only samples with detected miRNA were used for this analysis. A *p*-value of <0.05 was considered statistically significant.

## 3. Results

### 3.1. Description of Extracellular Vesicles

Samples of urine and plasma EVs, isolated by UC and the precipitation-based method, were characterized by transmission electron microscopy.

**Figure 2:**
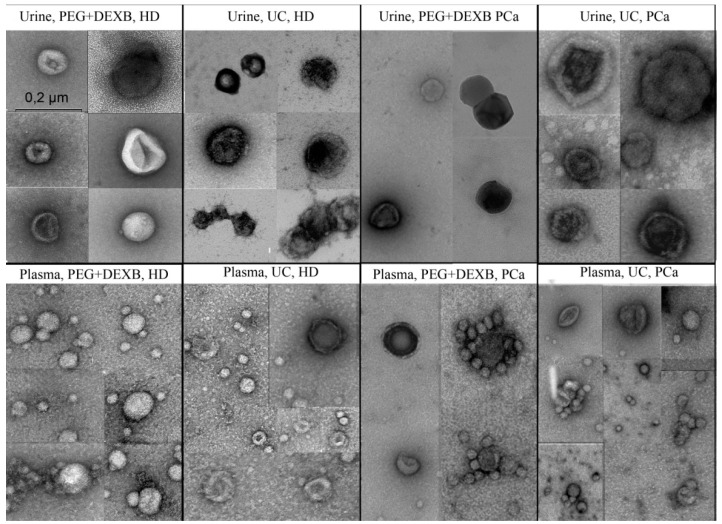
TEM images of plasma and urine extracellular vesicles of healthy donors and PCa patients obtained by precipitation using PEG and DEXB along with ultracentrifugation. The bar scale is provided for all presented TEM pictures.

According to TEM, EVs from the blood plasma and urine of healthy donors and PCa patients contained EVs mainly sized from 20 to 150 nm (Figure 2). Vesicles ranging from 30 to 150 nm (the exosome size range) were present in all analyzed samples (Figure 2).

In more detail, the size distribution of vesicles in the EV samples isolated from urine by both the UC and aggregation–precipitation-based methods are presented in Table 2 and Table 3. The majority of obtained EVs were sized 30–100 nm with the appearance of spherical bubbles or “cups” hinting at their exosomal/endocytic origin (typically exosomes). In samples isolated from the plasma by the aggregation–precipitation method, the exosome-like vesicles partly differed in appearance from typical exosomes in the form of rounded globular structures with dark-colored membranes. Their diameters varied mainly from 30 to 100 nm (Table 2 and Table 3), and sometimes the vesicles adhered together. The UC EV samples also contained such exosome-like vesicles together with typical exosomes. Exosomes with compacted (electron-dense) contents adjacent to the membrane, as well as defects in the integrity of the membrane and the appearance of inclusions within, were revealed in the EV samples obtained from the urine of PCa patients by both UC and the aggregation–precipitation-based method.

In the plasma EVs of PCa patients isolated via the aggregation–precipitation-based method, a significant densification of a part of the small (about 30 nm) exosome-like structures was observed. Moreover, typical exosomes with electron-dense contents under the membrane were revealed. The same tendency was observed to a lesser degree in the UC-isolated plasma EVs of the PCa patients. The mass adhesion of exosome-like vesicles and their frequent circular adhesion to the typical exosomes were detected in the EVs of the PCa patients isolated by both methods. 

**Figure 3:**
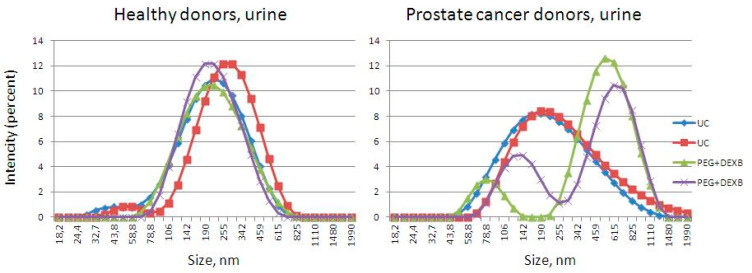
The size distribution of urine EVs isolated by precipitation (PEG+DEXB) and ultracentrifugation (UC), visualized by Zetasizer Nano ZS.

The widths of the peaks obtained from the urine EVs, isolated by the new precipitation protocol using the Zetasizer Nano ZS Plus, corresponded to 150–300 nm. In some samples, there were additional peaks observed at 10 and 70–100 nm (Figure 3). The widths of the peaks obtained from the urine EVs, isolated by ultracentrifugation, corresponded to 180–260 nm (Figure 3). The status of the donor did not have a significant impact on the EV isolation procedures.

**Figure 4:**
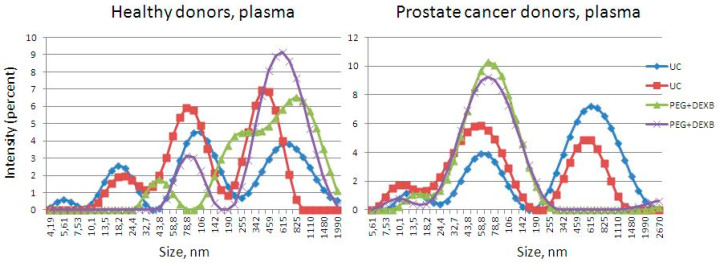
The size distribution of plasma extracellular vesicles isolated by precipitation (PEG+DEXB) and ultracentrifugation (UC), visualized by a Zetasizer Nano ZS.

The widths of the peaks obtained from the plasma EVs, isolated by the new precipitation protocol in the Zetasizer Nano ZS Plus, corresponded to 50–200 nm (Figure 4). Several peaks were obtained from the plasma EVs isolated by ultracentrifugation, corresponding to 20, 50–100, and 400–500 nm. Along with the exosomes and microvesicles, precipitation and UC resulted in the co-isolation of small (likely lipoproteins of low (18–26 nm) or high (8–15 nm) density) and large particles (likely apoptotic bodies and vesicles or particle aggregates), which is consistent with the data obtained by TEM. This was especially evident for the UC-based EV isolation from plasma.

### 3.2. Differential Efficiency of Isolation Methods for the Detection of miRNAs by RT-qPCR

**Figure 5:**
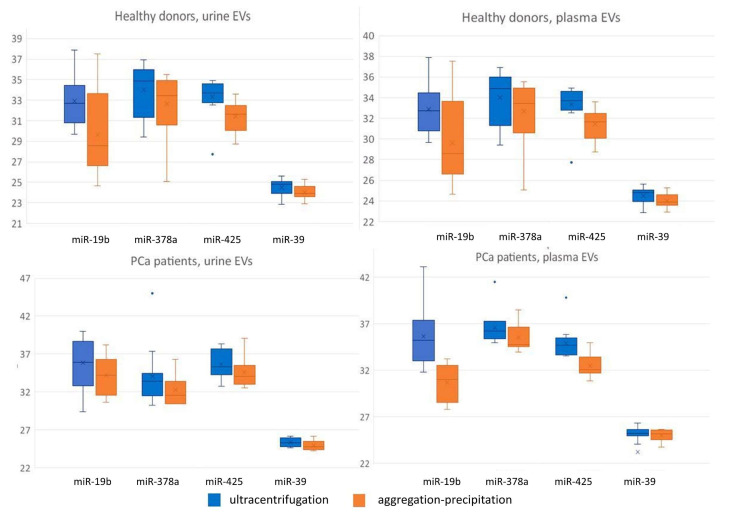
Mir-19b, miR-378a, miR-425, and cel-miR-39 Cp values isolated from the urine and plasma extracellular vesicles of healthy donors and PCa patients.

We investigated the performance of UC and precipitation protocols in the downstream RT-qPCR experiments for miRNA detection using a fixed volume of eluted RNA as input for the RT reaction. Factorial ANOVA statistics revealed a significant effect of the EV isolation method on the miR-19b (F (1,87) = 13.9, *p* < 0.001) and miR-425 (F (1,87) = 12.4, *p* < 0.001) Cp values. With a fixed RNA volume, UC and the precipitation protocols resulted in the equal detection of miR-19b, miR-378a, and miR-425 in urine and miR-378a in plasma EVs, whereas precipitation-based EV isolation yielded significantly higher miR-19b and miR-425 Cp values (*p* < 0.01) than UC, when isolated from plasma (Figure 5). All studied RNA samples produced Cp values within the working range of the systems. Spike-in control (cel-miR-39) is also illustrated in Figure 5.

The statistical analysis demonstrated a significant effect of donor status on the miR-19b (F (1,87) = 8.1, *p* < 0.01), miR-378a (F (1,87) = 19.6, *p* < 0.001), and miR-425 (F (1,87) = 26.9, *p* < 0.001) Cp values. UC EV isolation from the urine of healthy donors yielded significantly lower miR-19b (*p* < 0.01), miR-378a (*p* < 0.01), and miR-425 (*p* < 0.001) Cp values compared to the prostate cancer donors (Figure 5). The miR-425 Cp value of the precipitation-based isolated urine EVs of healthy donors decreased at a marginally significant level (*p* = 0.06) compared with that of the prostate cancer donors (Figure 5). In the plasma EVs, isolated by both UC and the precipitation-based protocol, mir-378a was detected at higher levels in the healthy donors EVs compared to the prostate cancer donors (*p* < 0.01, Figure 5). The miR-425 Cp values of both the UC and precipitation-based isolated plasma EVs of the healthy donors decreased at a marginally significant level (*p* = 0.06) compared to the prostate cancer donors (Figure 5).

**Figure 6:**
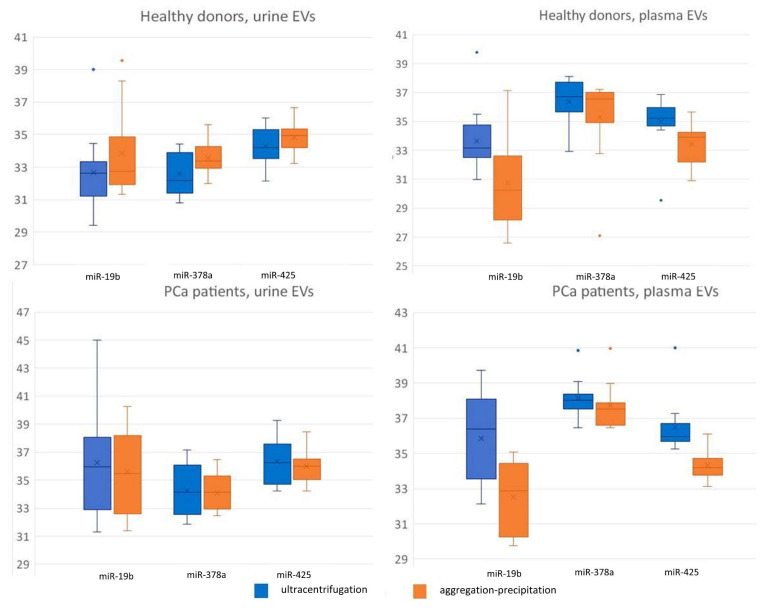
Mir-19b, miR-378, miR-425, and cel-miR-39 Cp values when isolated from the urine and plasma extracellular vesicles of healthy donors and PCa patients, five-fold diluted samples.

Factorial ANOVA statistics revealed a significant effect of the biological fluid type on mir-19b (F (1,87) = 5.8, *p* < 0.05), miR-378a (F (1,87) = 52.2, *p* < 0.001), and miR-425 (F (1,87) = 15.3, *p* < 0.001). A significant interaction between the isolation method and type of biological fluid for the miR-19b (F (1,87) = 5.3, *p* < 0.05) and miR-425 (F (1,87) = 8.4, *p* < 0.01) Cp values was also demonstrated. Both the UC and precipitation-based EV isolation from the urine of healthy and prostate cancer donors yielded significantly lower miR-378a Cp values (*p* < 0.01) compared to the plasma EVs (Figure 5). Moreover, the miR-425 precipitation isolated from urine EVs yielded significantly higher Cp values compared to the plasma EVs in both healthy and prostate cancer donors. The miR-425 Cp value of the UC-isolated urine EVs of healthy donors increased at a marginally significant level (*p* = 0.06) compared with the plasma EVs only for prostate cancer donors (Figure 5). An RT-PCR analysis of the urine and plasma EVs of healthy donors and PCa patients was conducted to assess the presence of PCR inhibitors in the analyzed samples. The statistical analysis demonstrated a significant effect of dilution on the miR-19b (F (1,81) = 168.0, *p* < 0.001), miR-378a (F (1,84) = 531.9, *p* < 0.001), and miR-425 (F (1,87) = 578.5, *p* < 0.001) Cp values (Figure 6). Significant interactions between the isolation method and dilution method and between the dilution and biological fluid type were also demonstrated for the miR-19b (F (1,81) = 34.3, *p* < 0.001), miR-378a (F (1,84) = 4.3, *p* < 0.05), and miR-425 F (1,87) = 5.6, *p* < 0.05) Cp values. 

Five-fold dilution of miRNA samples resulted in a significant increase in the Cp values of the miRNA in all investigated samples, except for the UC-isolated urine EVs of prostate cancer donors (Figure 6). This indicates that the UC-isolated urine EVs of the prostate cancer donor fraction contained the highest level of PCR inhibitors among all studied EV fractions. The only significant difference between the different EV isolation methods was observed for the plasma EV-diluted miR-425 (*p* < 0.01) Cp value (Figure 6), which remained higher in the EVs isolated by UC than the Cp values in the EVs isolated by the precipitation–aggregation protocol, as well as the Cp values in the undiluted EV samples.

The above-mentioned evidence all indicates that the developed precipitation–aggregation protocol is not inferior to UC for the isolation of urine EVs and is even a better choice for EV isolation from plasma.

## 4. Discussion

Current research into the discovery of EV-based biomarkers has induced great interest and a vast number of investigations in the fields of biology and medicine (for a review, see [25,30] and many others). However, there is still no consensus on what protocol should be used to isolate EVs for scientific and clinical use. Moreover, it is necessary to consider and optimize many technical factors before we can apply EVs in common clinical practice, as the methods used for EV isolation can significantly influence the final results [25].

**Figure 7:**
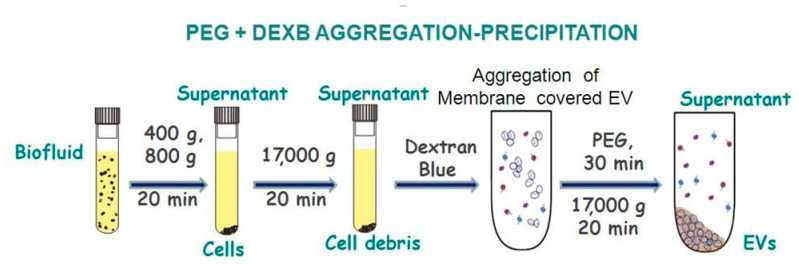
The scheme of the PEG- and DEXB-based precipitation of extracellular vesicles.

In this article, we designed an aggregation–precipitation-based method of EV isolation from plasma and urine samples for downstream miRNA analysis. The PEG-based EVs isolation is widely used. However, its main drawback is the co-precipitation of different polymers, it obstructs further analysis including miRNA analysis using RT-PCR. To make the isolation procedure more specific for membrane coated vesicles the Dextran Blue was used. In the first stage, a sample of biological fluid was subjected to low-speed centrifugation to reduce its contamination with non-vesicular particles (cells, cellular debris, apoptotic bodies, etc.). After this sample preparation, 50 min was required to isolate EVs using the novel precipitation-based method (for comparison, the ultracentrifugation time is 3 h). The subsequent addition of a NaCl solution to the supernatant made it possible to reduce the number of aggregates and exclude their coprecipitation during subsequent stages of EV isolation. In the next step of EV isolation, we added 1M TrisHCl pH = 7.0 to the supernatant to create optimal conditions for EV precipitation. Dextran Blue (2000 kDa) was used to initiate EV aggregation to precipitate membrane-coated particles more effectively than membrane-free microparticles, large biopolymers, and supramolecular complexes (Figure 7). The subsequent addition of PEG (20 kDa) facilitated effective EV precipitation via low-speed centrifugation. At the same time, the employed Dextran Blue and PEG concentrations were small enough not to cause precipitation of the polymers, including those inhibiting PCR. 

In this article, we designed an aggregation–precipitation-based method of EV isolation from plasma and urine samples for downstream miRNA analysis. The PEG-based EVs isolation is widely used. However, its main drawback is the co-precipitation of different polymers, it obstructs further analysis including miRNA analysis using RT-PCR. To make the isolation procedure more specific for membrane coated vesicles the Dextran Blue was used. In the first stage, a sample of biological fluid was subjected to low-speed centrifugation to reduce its contamination with non-vesicular particles (cells, cellular debris, apoptotic bodies, etc.). After this sample preparation, 50 min was required to isolate EVs using the novel precipitation-based method (for comparison, the ultracentrifugation time is 3 h). The subsequent addition of a NaCl solution to the supernatant made it possible to reduce the number of aggregates and exclude their coprecipitation during subsequent stages of EV isolation. In the next step of EV isolation, we added 1M TrisHCl pH = 7.0 to the supernatant to create optimal conditions for EV precipitation. Dextran Blue (2000 kDa) was used to initiate EV aggregation to precipitate membrane-coated particles more effectively than membrane-free microparticles, large biopolymers, and supramolecular complexes (Figure 7). The subsequent addition of PEG (20 kDa) facilitated effective EV precipitation via low-speed centrifugation. At the same time, the employed Dextran Blue and PEG concentrations were small enough not to cause precipitation of the polymers, including those inhibiting PCR. 

To develop the protocol described above, several precipitating solutions were first tested to reveal the optimal concentrations of components (Table S1). Briefly, higher PEG concentrations resulted in higher Cp values, whereas higher Dextran Blue concentrations inhibited PCR and resulted in undetected Cp values. Then, we compared the novel protocol for EV enrichment with ultracentrifugation to assess which is better suited for miRNA profiling.

Despite the large number of studies devoted to the development of new protocols for EV isolation and the comparison of different methods with each other, there are only a limited number of articles comparing the expression of specific EV RNAs (and miRNA in particular) isolated by different methods (for example [31,32,33,34]). At the same time, miRNAs are promising markers for different diseases. EV miRNAs used as biomarkers do not demand the isolation of specific EV subpopulations as quickly; regardless of their origin, all EVs transport biomolecules, including RNA (in particular, miRNA) [8,35,36]. Therefore, the main drawbacks of EV isolation based on PEG precipitation (i.e., the isolation of a mixture of extracellular vesicles of different types and the aggregation and retention of the polymer [37,38]) do not negatively influence miRNA analysis, except through the precipitation of protein aggregates and supramolecular complexes by PEG in the concentration proposed for EV isolation. Precipitation methods in general, and the aggregation–precipitation method in particular, are poorly suited for microscopic studies because of membrane fusion (PEG induce membrane fusion used for hybridoma production [39] and micromolecule precipitation, for example, 5–7% PEG also precipitates immune complexes, cryoglobulines, and complement components [40,41]). Precipitating reagents may give a very high background signal, hampering analysis of the samples [37]. Nevertheless, TEM revealed strong similarities between the EV samples isolated by UC and precipitation. All EVs contained exosome-like vesicles according to their size and shape. Plasma EVs isolated by both methods, along with exosomes, contained small exosome-like vesicles, which may—at least partially—refer to lipoproteins. Noteworthy samples obtained from the PCa samples by both methods were characterized by EV abnormalities, such as adhesion, the presence of exosomes with a compaction of the content adjacent to the membrane, defects in the integrity of the membrane, and inclusions inside the EVs. These abnormalities may be due to lipid metabolism disturbances related to the development of oncological disease [42,43].

In the plasma EVs of PCa patients isolated by the aggregation–precipitation-based method, a significant densification of a part of the small (about 30 nm) exosome-like structures was observed. Moreover, typical exosomes with electron dense contents under the membrane were revealed. The same tendency, albeit to a lesser degree, was observed in the UC-isolated plasma EVs of PCa patients. The mass adhesion of exosome-like vesicles and their frequent circular adhesion to typical exosomes were detected in the EVs of PCa patients, isolated by both methods. 

Moreover, it was shown that this novel precipitation-based method allows quick and effective EV isolation from biological fluids. With a fixed RNA volume, UC and precipitation protocols resulted in the equal detection of miR-19b, miR-378a, and miR-425 in urine and miR-378a in plasma EVs, whereas precipitation-based EV isolation from plasma yielded significantly higher miR-19b and miR-425 than UC. The significantly higher Cp values suggest a lower recovery of EVs and/or poor PCR efficiency due to nonvesicular contamination. The latter is also supported by the data of the RT-qPCR analysis of diluted miRNA, which indicated the presence of a greater amount of PCR inhibitors in the miRNA of EVs after differential ultracentrifugation (Figure 6). The higher yield of miR-19b and miR-425 in the EVs after precipitation-based isolation than after ultracentrifugation and the equal detection of miR-378 in plasma EVs isolated by different methods indicate that EVs containing certain miRNA may be selectively enriched when isolated by a certain method. Such selectivity occurred in detailed studies of the methods for EV isolation [37] and could be associated with the degree of encapsulation of each individual miRNA, since it is an intrinsic miRNA characteristic. Most likely, the use of a certain urine (or other biological fluid) fraction depends on an analysis of a particular miRNA, since the expression of miRNAs may differ between fractions in various ways [10,44,45]. However, the results obtained point to precipitation based EVs isolation as suitable for efficient enrichment of EVs for subsequent miRNA analyses, which is supported by previous studies [37,46].

One of advantage of the method developed in this work is that the present method can be modified for various biological fluids. In this article, we showed that this method can be used for EV miRNA isolation from both blood and urine (with slight modifications). The isolation of EVs from various biological fluids facilitates the development of highly sensitive diagnostics of diseases of different organs, since the EVs containing the greatest numbers of biomarkers are mostly represented in biological fluid directly in contact with the parental cells. For example, the most suitable biological fluid for microparticle isolation when searching for the biomarkers of lung cancer is bronchoalveolar lavage, while when studying kidney, prostate, or bladder cancer, it is better to use urine [47,48,49,50]. Urine, in contrast to blood plasma, contains a higher concentration of salts and is highly diluted (in its content of extracellular EVs), as well as characterized by a lower concentration of potential biomarkers and a wider pH and osmolarity range. Moreover, urine contains the Tamm–Horsfall protein, which significantly complicates EV isolation [51]. These features lead to some challenges in EV isolation from this biological fluid. Thus, most EV isolation methods are poor for diluted biofluids. In response, scientists have developed method modifications (ExoQuick-TC method: [52]) or specific isolation methods for urine EVs, such as hydrostatic dialysis [53]. The reason for the variability in the effectiveness of EV miRNA detection in different donors and the absence of significant differences of miRNA detection in urinary EVs isolated by different methods could be a change in the composition and concentration of urine depending on the patient’s diet and medications taken.

To date, ultracentrifugation remains the most commonly used method for EV isolation from different biological fluids. A systematic analysis of the relevant literature demonstrates that 90% of the studies on EV isolation conducted before 2015 utilized ultracentrifugation [54]. Along with its advantages, ultracentrifugation has some significant limitations. The procedure is time-consuming, highly labor-intensive, requiring expensive equipment, gives poor results, and has very low reproducibility, likely due to a great variability in resuspension of the EV pellet [37,55,56]. This creates significant challenges for the routine clinical use of UC. Among other traditional methods used for EV isolation, there are microfiltration and gel filtration. Methods based on EVs changing their solubility and/or aggregating appeared somewhat later and included precipitation with polyethylene glycol, protamine, and sodium acetate. Protamine, a positively charged molecule, is used to aggregate and isolate EVs as soon as they are negatively charged. The disadvantage of this method is its co-isolation of other negatively-charged particles. Co-isolated protamine and lipoproteins influence subsequent analysis, so purification of the isolated fraction from protamine and lipoproteins via heparin and gel filtration is necessary [57]. EV fractions isolated by sodium acetate can also be contaminated by non-EV proteins [58]. In addition, numerous methods for isolating the EV population based on highly specific interactions with the molecules exposed on the EV surface or using microfluidic technologies have recently appeared (for a review, see [25]). Today, these methods for EV isolation are becoming more frequently used. Thus, the share of the papers involving EV isolation via ultracentrifugation published during 2014–2017 decreased to 62.1%, even among those using ultracentrifugation as a control [25]. 

Overall, the development and comparison of existing EV isolation methods has attracted significant attention due to the potential application of EVs in clinical practice, including EV miRNA analysis [31,37,52]. For example, Andreu et al. conducted a comparative analysis of six different EV isolation procedures for miRNA detection in serum samples. Different methods based on precipitation, columns, or filter systems were tested and compared via ultracentrifugation. Overall, the precipitating reagents gave a better yield than other commercial kits. The EVs isolated via precipitation-based methods were enriched in miR-126, miR-30c, and miR-143, while the detection of miR-21, miR-16-5p, and miR-19a was the same. The overall performance of PEG was very similar to that of commercial precipitating reagents (ExoQuick-TM, System Biosciences, United States and Total exosome isolation precipitating agent; Invitrogen by Life Technologies, United States) in both protein and miRNA yield, but PEG is much cheaper [37]. Wachalska et al. compared the effectiveness of ultracentrifugation, a Life Technologies EV isolation kit (#4484452 Life Technologies, Mulgrave, VIC, Australia), and a NorgenBiotek urine EV RNA isolation kit (#47200, NorgenBiotek, Thorold, ON, Canada). Contrary to the previous study, it was shown that the miRNA levels in the EVs after UC were higher compared to those in both commercial kits [31]. 

Some other authors assessed the total RNA and miRNA amounts in the EVs under different isolation techniques. For example, Alvarez et al. used six different protocols; three were based on ultracentrifugation, one used a nanomembrane concentrator-based approach, and two utilized a commercial exosome precipitation reagent (ExoQuick-TC; System Biosciences) [52]. The highest yield of exosomes was obtained using a modified exosome precipitation protocol, which also yielded the highest quantities of miRNA and mRNA. The following differences were observed between the standard and modified ExoQuick-TC-based methods: the addition of DL-dithiothreitol, a larger volume of ExoQuick-TC reagent, and a higher final centrifugation speed in the modified protocol [52]. When exosomes were isolated from ascite specimens, it was shown that, while each method purifies exosomal material, the circulating exosomes isolated by ExoQuick precipitation produced exosomal RNA and protein with greater purity and quantity than those isolated via chromatography, ultracentrifugation, and DynaBeads [59]. Buschmann et al. [33] compared the suitability of several EV isolation methods for miRNA analysis and miRNA biomarker discovery. The authors showed that isolation via precipitation and membrane affinity is more suitable for miRNA-based biomarker discovery than size-exclusion chromatography, which failed to separate patients from healthy volunteers [33]. The commercial kits for polymer based EVs precipitation are widely used. In comparison with these kits the PEG+DEXB based method is very cheap, while the labor intensiveness is the same. The time of the isolation by commercial kits is usually over 45–65 min (see [25] for review), sometimes with overnight incubation. From this point of view the developed method is also not inferior to commercial kits for EVs isolation. Thus, a comparison between the published reports and the obtained data led to the conclusion that precipitation-based protocols for EV isolation are effective and useful for subsequent miRNA analysis in different biological fluids. However, it should be noted that the developed method, as well as other protocols for polymer based EVs precipitation, UC, ultrafiltration and the range of other methods allow nonspecific isolation of EVs from biofluids, whereas the EVs enrichment through immune-affinity and peptide-affinity may isolate tumor exosomes specifically [60] and potentially detect tumor-specific molecules more sensitively.

## 5. Conclusions

In summary, the precipitation method described in this work allows quick and effective EV isolation from plasma and urine. This method is characterized by several advantages: It is fast and simple, it does not need complicated equipment, it can be modified for different biological fluids, it has a low cost, and it can simultaneously process a large number of samples and isolate EVs in an amount sufficient for miRNA analysis. Taken together, these facts make the precipitation method of EV isolation accessible and suitable for both research and clinical laboratories. Moreover, the developed method has a great potential to increase the diagnostic and prognostic utilization of EVs from blood plasma and urine including miRNA-based diagnostics of urolgenital pathologies.

## 6. Patents

Russian patent Konoshenko MY, Lekchnov EA, Bryzgunova OE, Laktionov PP. Method of extracellular vesicles isolation from biological fluids. RF patent no. 2678988, 5 February 2019.

## Figures and Tables

**Table 1:** Sequences of primers and probes used for reverse transcription and TaqMan qPCR.

miRNA	Label	Sequence
	Universal Reverse Primer	5′-GTGCAGGGTCCGAGGT-3′
hsa-miR-19b-3p	RT	5′-GTCGTATCCAGTGCAGGGTCCGAGGTATTCGCACTGGATACGACTCAGTT-3′
(miR-19b)	Forward	5′-CGCTGTGCAAATCCATGCAA-3′
	Probe	5′-(FAM)-GCACTGGATACGACTCAGTT-(FQ)-3′
hsa-miR-378a-3p	RT	5′-GTCGTATCCAGTGCAGGGTCCGAGGTATTCGCACTGGATACGACGCCTTC-3′
(miR-378)	Forward	5′-GACTGGACTTGGAGTCA-3′
	Probe	5′-(FAM)-CGCACTGGATACGACAAAGTC-(BHQ)-3′
hsa-miR-425-5p	RT	5′-GTCGTATCCAGTGCAGGGTCCGAGGTATTCGCACTGGATACGACTCAACG-3′
(miR-425)	Forward	5′-TAATGACACGATCACTCC-3′
	Probe	5′-(FAM)-CGCACTGGATACGACTCAACG-(BHQ)-3′
cel-miR-39-3p	RT	5′-GTCGTATCCAGTGCAGGGTCCGAGGTATTCGCACTGGATACGACCAAGCT-3′
(miR-39)	Forward	5′-ATTCACCGGGTGTAAATC-3′
	Probe	5′-(FAM)-CACTGGATACGACCAAGCTGA-(BHQ)-3′

**Table 2:** The TEM data on EVs size in samples from healthy donors (HD). Percentage and median size is presented. The percentage of EVs (from 31 to 250 nm) was counted from total number of particles in the sample (0–250 nm). The percentage of EVs fractions (from 31 to 50 nm, from 51 to 100 nm, from 101 to 150 nm, from 151 to 200 nm, from 201 to 250 nm) were counted from number of EVs ranged from 30 to 250 nm (considered as 100%).

HD	nm	31–250	31–50	51–100	101–150	151–200	201–250
PlasmaUC	%	71	82	16	2	0	0
	median, nm	45	41	55	133		
PlasmaPEG+DEXB	%	86	67	32	1	0	0
	median, nm	47	45	56	119		
UrineUC	%	100	5	67	28	0	0
	median, nm	89	43	80	119		
UrinePEG+DEXB	%	100	11	65	18	4	2
	median, nm	70	41	68	117	174	240

**Table 3:** The TEM data on EVs size in samples from PCa patients (PCa). Percentage and median size is presented. The percentage of EVs (from 31 to 250 nm) was counted from total number of particles in the sample (0–250 nm). The percentage of EVs fractions (from 31 to 50 nm, from 51 to 100 nm, from 101 to 150 nm, from 151 to 200 nm, from 201 to 250 nm) were counted from number of EVs ranged from 30 to 250 nm (considered as 100%).

PCa	Range, nm	31–250	31–50	51–100	101–150	151–200	201–250
PlasmaUC	%	53	91	7	2	0	0
	median, nm	40	39	56	109		
Plasma PEG+DEXB	%	56	86	14	0	0	0
	median, nm	40	38	59			
UrineUC	%	96	13	47	23	10	7
	median, nm	93	47	81	126	178	222
Urine PEG+DEXB	%	86	9	64	21	5	1
	median, nm	78	34	71	124	171	211

## Data Availability

The data presented in this study are available on request from the corresponding author.

## Supplementary Materials

The following are available online at https://www.mdpi.com/2075-4418/11/3/384/s1, Table S1: The effectiveness of miRNA isolation from urine EVs obtained by precipitation and aggregation-precipitation approaches with different composition of solution.
