# Supplementary material for: Isolation of Extracellular Vesicles from Biological Fluids via the Aggregation–Precipitation Approach for Downstream miRNAs Detection
Source: Diagnostics (Basel). 2021 Feb 24;11(3):384. doi: 10.3390/diagnostics11030384 (PMC7996260; doi:10.3390/diagnostics11030384)
Supplement: Supplementary file 1 [file diagnostics-11-00384-s001.zip › diagnostics-1098440-supplementary.pdf]

diagnostics

MDPI

Table s1

Isolation solution	ΔCp = Cp (of the method)- Cp (of final aggregation-precipitation approach)										mean ΔCp	ΔCpSD
	1*	2*	3*	4*	5*	6*	7*	8*	9*	10*		
**$PEG_{6,000}$ 10%**	5,23	4,23	4,46	4,45	4,61	4,48	4,42	5,50	5,02	4,84	4,72	0,40
**$PEG_{20,000}$ 5%**	3,00	2,47	3,43	2,56	2,63	3,07	3,39	3,00	2,70	2,60	2,87	0,22
**$PEG_{20,000}$ 1,5%**	3,09	3,08	3,40	2,24	2,27	2,84	3,23	2,58	3,14	2,63	2,86	0,44
**DEX Blue 4µg/ml, $PEG_{20,000}$ 1,5%**	2,58	2,13	2,03	2,49	2,47	2,04	1,69	2,24	1,96	2,22	2,15	0,34
**TrisHCl 50mM, DEX Blue 4µg/ml , $PEG_{20,000}$ 1,5%**	1,41	1,43	1,42	1,51	1,01	1,13	2,06	1,46	1,38	1,49	1,44	0,25

Table s1. The effectiveness of miRNA isolation fromurine EVs obtained by precipitation and aggregation-precipitation approaches with different composition of solution. The table shows the relative expression of miRNA-19b in EVs obtained by the above methods and final aggregation-precipitation approach (ΔCp). * - donor number.
